# HPV and cofactors for invasive cervical cancer in Morocco: a multicentre case-control study

**DOI:** 10.1186/s12885-017-3425-z

**Published:** 2017-06-20

**Authors:** Mohamed Berraho, Afaf Amarti-Riffi, Mohammed El-Mzibri, Rachid Bezad, Noureddine Benjaafar, Abdelatif Benideer, Noureddine Matar, Zinab Qmichou, Naima Abda, Mohammed Attaleb, Kaoutar Znati, Hind El Fatemi, Karima Bendahhou, Majdouline Obtel, Abdelhai Filali Adib, Simone Mathoulin-Pelissier, Chakib Nejjari

**Affiliations:** 1 Laboratory of Epidemiology, Clinical Research and Community Health, Faculty of Medicine and Pharmacy, University Sidi Mohammed Benabdellah, BP.1893, Km 2.2 Route Sidi Harazem, Fez, Morocco; 2 Pathological Anatomy Laboratory, CHU Hassan-II, Fez, Morocco; 3 Biology Unit and Medical Research, National Center of Energy, Sciences and Nuclear Techniques (CNESTEN), Rabat, Morocco; 4 Hospital “Les Orangers”, CHU IbnSina, Rabat, Morocco; 5 Faculty of Medicine and Pharmacy, University Mohammed V Souissi, Rabat, Morocco; 6 Department of Radiotherapy, National Institute of Oncology, Rabat, Morocco; 7 Department of Oncology-Radiotherapy, CHU IbnRochd, Casablanca, Morocco; 8 Department of Gynecology and Obstetrics, CHU IbnRochd, Casablanca, Morocco; 9 Faculty of Science and technology, Mohammedia, Morocco; 10 Cancer Registry of Casablanca, Casablanca, Morocco; 11 Directorate of Epidemiology and Fight against Disease (DELM) Ministry of Health, Rabat, Morocco; 12 Univ. Bordeaux, ISPED, Centre INSERM U897 – Epidemiologie-Biostatistique, F-33000 Bordeaux, France; 13 CIC-EC7, Clinical and Epidemiological Research Unit, Institut Bergonié, Comprehensive Cancer Centre, Bordeaux, France

**Keywords:** Cervical cancer, Hpv, Determinants, Case-control study, Morocco

## Abstract

**Background:**

Limited national information is available in Morocco on the prevalence and distribution of HPV-sub-types of cervical cancer and the role of other risk factors. The aim was to determine the frequency of HPV-sub-types of cervical cancer in Morocco and investigate risk factors for this disease.

**Methods:**

Between November 2009 and April 2012 a multicentre case-control study was carried out. A total of 144 cases of cervical cancer and 288 age-matched controls were included. Odds-ratios and corresponding confidence-intervals were computed by conditional logistic regression models.

**Results:**

Current HPV infection was detected in 92.5% of cases and 13.9% of controls. HPV16 was the most common type for both cases and controls. Very strong associations between HPV-sub-types and cervical cancer were observed: total-HPV (OR = 39), HPV16 (OR = 49), HPV18 (OR = 31), and multiple infections (OR = 13). Education, high parity, sexual intercourse during menstruation, history of sexually transmitted infections, and husband’s multiple sexual partners were also significantly associated with cervical cancer in the multivariate analysis.

**Conclusions:**

Our results could be used to establish a primary prevention program and to prioritize limited screening to women who have specific characteristics that may put them at an increased risk of cervical cancer.

## Background

Cervical cancer is the second most common cancer among women worldwide, with an estimated 529,409 incident cases and 274,883 deaths in 2008 [1]. More than 80 % of new cases are currently diagnosed in developing countries [2].

Some sub-types of human papillomavirus (HPV) are the central and necessary cause of cervical cancer [3]. HPV infection appears to be a necessity but not a sufficient cause of cervical cancer [3]. The models of cervical pathogenesis involve persistent infection caused by high-risk HPV as well as cofactors that increase the risk of cervical cancer.

In Morocco, cervical cancer is the second most common cancer for women [4]. In 2008, the world age-standardized incidence of cervical cancer among women in Morocco was 14.1/100000 inhabitants/year [4] and the mortality rate was 8.4/100000 (1152) [2].

There is a lack of information on the prevalence and distribution of HPV sub-types of cervical cancer in Morocco as well as on the role of other risk factors for cervical cancer [5–8]. In Morocco, only one single center case-control study focusing on invasive cervical cancer was carried out in 1993 [9]. It demonstrated that HPV was the central cause of more than 90% of the cervical cancer cases, however; some methodological limitations have been noted such as the lack of matching for age. Also, the role of specific viral hosts or other factors (sociodemographic, behavioral and genetic) in the progression from infection to invasive disease has not been clarified for Moroccan women.

To assess the role of HPV infection and other associated factors in the development of cervical cancer, we have carried out a case-control study of invasive cervical cancer in Morocco.

## Methods

### Study population

Between November 2009 and April 2012, we performed our case-control study in three cities of Morocco: Rabat, Casablanca and Fez. A total of 144 patients with cervical cancer and 288 controls were included. The cases were recruited in three centers: the National Institute of Oncology of Rabat, the Oncology center of the Ibn Roshd University Hospital of Casablanca and the gynecologic centers of Hassan-II University Hospital of Fez. Inclusion criteria for cases included having a newly diagnosed, histologically confirmed cervical cancer, and having no previous cervical cancer treatment.

Controls were selected from both public outpatient gynecological hospitals and primary health-care centers with gynecological units. They were selected using an individual age-matching (± 5 years). Exclusion criteria for controls included: diagnosis or history of cancer, history of hysterectomy, cervical conization or cervical cytologic abnormality.

### Data and specimen collection

Protocols were approved by Ethical Review Committees of the Fez Faculty of Medicine and the Hassan-II University Hospital. Because of the high illiteracy rate among this population, we have not been able to administer a written information sheet. Instead, an explanation of its content has been given to the patients and their verbal consent have been gathered.

A trained nurse administered a standardized questionnaire to all participants. The questionnaire items concerned the socio-demographic characteristics, sexual behavior, genital hygiene, history of sexually transmitted diseases, reproductive and contraceptive history and smoking.

All patients received a pelvic examination performed by an oncologist or a gynecologist. For the cases, a tumor biopsy specimen was taken for HPV-DNA detection and typing and it was performed by an oncologist. For the controls, cervical exfoliated cells were obtained and followed by PAP-smear preparation. Besides, cervical specimens were placed in tubes with PBS for HPV-DNA detection and typing.

### Detection and typing of HPV-DNA

The nested PCR-amplification of a conserved region of the HPV-L1 gene-DNA with the consensus MY09/MY11 and GP5+/GP6+ primers followed by genotyping with direct DNA-sequencing, generally accepted as a scientific tool in research [10, 11] was used in our study.

For DNA-sequencing, the nested PCR products, if positive, were purified by PCR purification ExoSaP-IT clean-up system (USB-USA) and sequenced directly using GP6+ primer as the sequencing primer and BigDye®Terminator v3.1 Cycle Sequencing Kit (AppliedBiosystems, Foster-city, CA-USA), according to manufacturer’s protocol, on an ABI3130XL-DNA analyzer. Nucleotides sequences were aligned and compared with those of known HPV-sub-types available through GenBank by using the online BLAST 2.0 software server (https://www.ncbi.nlm.nih.gov/blast/).

### Statistical analysis

First off, we describe the cases and controls according HPV infections and other factors. As age is an individual matching variable, for all other factors Odds-ratios and 95% confidence intervals were calculated by means of conditional logistic regression.

In our bivariate analysis, we calculated adjusted OR for residence area and HPV infection (using a conditional logistic regression). In our population, the distribution of cases and controls by residence area was different. Thus, we consider it as a major confounding variable.

To assess the role of factors, other than HPV, we used a multivariate conditional logistic regression with reduction strategy. All variables statistically associated to cervical cancer in bivariate analysis, with the exception of HPV infection and residence area, were used in the multivariate analysis. *P* = 0.05 was the level of statistical significance. Statistical analyses were performed with SAS® software 9.1.

## Results

The mean age of the cases was 51.9 ± 11.9 years while the mean age of the controls 51.3 ± 12.6 (*p* = 0.8). Among 144 cases, 96.4% were squamous cell carcinoma and 4.7% were adeno/adenosquamous carcinoma.

Diagnosis of the controls in the study was distributed as follows: gynecologic diseases 17.0%, hypertension 13.8%, gastrointestinal diseases 10.9%, pelvic and other pain 10.6%, general symptoms (fever, headache, asthenia....) 9.2%, pregnancy control 8.1%, diabetes 7.1%, rheumatology diseases 6.0%, respiratory diseases 4.2%, dermatologic diseases 3.9%, and other diagnosis 9.2%.

### HPV infection

HPV infection was detected in 92.5% of all cases (92.2% of squamous cell carcinomas and 100% of adenocarcinomas) and in 13.9% of control women. Eight different HPV sub-types were identified in either single or multiple infections. HPV 16 was by far the most common type among cases as well as control women. Only HPV 16 infections were found among the 6 adenocarcinoma cases. Four controls and no cases were infected with low-risk HPV sub-types (Table 1).

**Table 1:** Distribution of HPV infection by HPV type and multiplicity of infection among 133 cases of cervical cancer and 281 controls^a^

	Total Cases		Squamous cell carcinoma		Adenocarcinoma		Controls	
	No.	(%)	No.	(%)	No.	(%)	No.	(%)
Total HPV tested	133	100	116	100	6	100	281	100
HPV Negative	10	7.5	9	7.8	-	-	242	86.1
HPV Positive	123	92.5	107	92.2	6	100	39	13.9
High risk HPB^b^	123	92.5	107	92.2	6	100	35	12.5
Low risk HPV^b^	-	-	-	-			4	1.4
HPV types								
Single infection	118	88.7	102	87.9			33	11.7
1	-	-	-	-	-	-	2	0.7
6	-	-	-	-	-	-	1	0.4
11	-	-	-	-	-	-	1	0.4
16	108	81.2	92	79.2	6	100	14	5.0
18	7	5.3	7	6.0	-	-	1	0.4
31	1	0.8	1	0.9	-	-	-	-
33	1	0.8	1	0.9	-	-	14	5.0
35	1	0.8	1	0.9	-	-	-	-
Multiple infection	5	3.8	5	4.3	-	-	6	2.2
16/31	4	3.0	4	3.4	-	-	-	-
16/33	1	0.8	1	0.9	-	-	5	1.8
16/31/33	-	-	-	-	-	-	1	0.4

^a^HPV information not available for 11 cases and 7 controls; ^b^High-risk types (HPV 16, 18, 31, 33 and 35). Low-risk HPV: (1, 6, 11)

The odds of cervical cancer was 39.3 times higher (95% CI: 16.0–96.5) for women with HPV infection of any type compared to women without HPV (Table 2). The odds of cervical cancer was 49.3 times higher (95% CI: 19.2–407.0) for women with HPV 16 infection compared to women without HPV (Table 2). The odds of cervical cancer was 31.7 times higher (95% CI: 2.5–407.0) for women with HPV 18 infection compared to women without HPV (Table 2).

**Table 2:** HPV infection among 133 cases of cervical cancer and 281 controls

	Cases		Controls		OR^a^ (95% CI)
	No.	(%)	No.	(%)	
HPV					
Negative	10	7.5	242	86.1	1
Positive (any type)	123	92.5	39	13.9	39.3 (16.0–96.5)
Multiple infection					
No	118	95.9	33	84.6	1
Yes	5	4.1	6	15.4	0.2 (0.02–1.7)
HPV type					
Negative	10	7.5	242	86.1	Reference
16	108	81.2	14	5.0	49.3 (19.2–126.3)
18	7	5.3	1	0.4	31.7 (2.5–407.0)
HPV 1, 6, 11	0	0.0	4	1.5	<0.001 (<0.001–999)
30s (31, 33, 35)	3	2.4	14	5.0	1.6 (0.4–6.9)
Multiple infection	5	3.8	6	2.2	13.8 (2.6–73.8)

^a^OR: Non-adjusted Odd Ratio

### Sociodemographic and socioeconomic characteristics

In the bivariate analysis (Table 3), an increased risk for cervical cancer was found among separated or divorced women (OR vs. married women =2.4). Non-educated women (OR vs. educated =4.7), absence of health insurance (OR = 3.8), residence in rural area (OR = 10.7) and low socioeconomic level (OR = 3.1 vs. middle and high). After adjustment for residence area and HPV infection, only non-educated women (OR vs. educated women =3.4), absence of health insurance (OR = 3.1), and low socioeconomic level (OR = 2.2) increased the risk of cervical cancer.

**Table 3:** Demographic and socio-economic characteristics among 144 cases of cervical cancer and 288 controls

	Cases		Controls		OR^a^ (95% CI)	ORa^b^ (95% CI)
	No.	(%)	No.	(%)		
Marital status	*n* = 144		*n* = 288			
Married	86	59.7	209	72.6	1	1
Widowed	36	25.0	58	20.1	1.7 (0.9–3.0)	1.5 (0.7–2.9)
Separated or divorced	22	15.3	21	7.3	2.4 (1.3–4.6)	1.6 (0.7–3.6)
Health insurance	*n* = 138		*n* = 283			
Yes	20	14.5	114	40.3	1	1
No	118	85.5	169	59.7	3.8 (2.2–6.6)	3.1 (1.7–5.5)
Residence area	*n* = 143		*n* = 271			
Urban and sub-urban	85	59.4	251	92.6	1	
Rural	58	40.6	20	7.4	10.7 (5.3–21.8)	
Education	*n* = 134		*n* = 273			
Educated	21	15.7	118	43.2	1	1
Non-educated	113	84.3	155	56.8	4.7 (2.6–8.4)	3.4 (1.1–11.0)
Socioeconomic level	*n* = 133		*n* = 279			
Middle and high	22	16.5	102	36.6	1	1
Low	111	83.5	177	63.4	3.1 (1.8–5.4)	2.2 (1.2–3.9)
Work outside home	*n* = 144		*n* = 288			
Never	131	91.0	245	85.1	1	1
Yes	13	9.0	43	14.9	0.6 (0.3–1.2)	0.7 (0.2–3.4)

^a^OR: Non-adjusted Odd Ratio

^b^ORa: Adjusted Odd Ratio for residence area and HPV infection

### Reproductive factors and contraceptive methods

In the bivariate analysis (Table 4), women between 13 and 14 years or ≥15 years at menarche had a higher risk than those who had an age at menarche ≤12 (OR = 3.6 and OR = 2.4 respectively). The number of pregnancies was also significantly associated with cervical cancer risk, where women with 4 and more pregnancies had a higher risk than those who had ≤3 pregnancies (OR = 1.4). Age at first pregnancy between 19 and 22 years (vs. ≤18 years) increased the risk of cervical cancer (OR = 2.2). Women reaching menopause at <45 years and between 45 and 49 years, compared to ≥50 years, was associated with an OR of 3.3 and 3.2 respectively. Use of oral contraceptives ≥6 years was associated with increase cervical cancer risk (OR = 1.8). Women who had never used a condom had an increased risk for cervical cancer (OR = 3.3). After adjustment for residence area and HPV infection, all associations observed in the bivariate analyses still significant except association between number of pregnancy, age at menopause and cervical cancer.

**Table 4:** Reproductive factors, contraceptive methods and screening variables among 144 cases of cervical cancer and 288 controls

	Cases		Controls		OR^a^ (95% CI)	ORa^b^ (95% CI)
	No.	(%)	No.	(%)		
Age at menarche (in years)	*n* = 140		*n* = 257			
≤ 12	25	17.9	103	40.1	1	1
13–14	78	55.7	90	35.0	3.6 (2.1–6.2)	3.6 (1.9–6.9)
≥ 15	37	26.4	64	24.9	2.4 (1.3–4.4)	2.6 (1.3–5.5)
No. of pregnancies	*n* = 141		*n* = 276			
≤ 3	40	28.4	119	43.1	1	1
> 3	101	71.6	157	56.9	1.4 (1.1–2.0)	1.2 (0.7–2.2)
Age at first pregnancy	*n* = 130		*n* = 254			
≤ 18	52	40.0	99	39.0	1	1
19–22	55	42.3	60	23.6	2.2 (1.2–3.9)	2.2 (1.1–4.4)
> 22	23	17.7	95	37.4	0.4 (0.2–0.8)	0.4 (0.2–0.9)
Age at last pregnancy	*n* = 120		*n* = 228			
≤ 35	67	55.8	119	52.2	1	1
> 35	53	44.2	109	47.8	0.9 (0.6–1.5)	0.9 (0.5–2.3)
Menopause	*n* = 142		*n* = 278			
No	66	53.5	129	46.4	1	1
Yes	76	46.5	149	53.6	1.1 (0.6–2.2)	1.0 (0.5–2.9)
Age at menopause						
≥ 50	29	40.3	93	66.0	1	1
45–49	26	36.1	28	19.9	3.2 (1.3–7.6)	4.2 (0.9–16.5)
< 45	17	23.6	20	14.2	3.3 (1.2–9.2)	2.5 (0.8–7.9)
Years of use of oral contraceptives	*n* = 140		*n* = 277			
< 6	85	60.7	201	72.6	1	1
≥ 6	55	39.3	76	27.4	1.8 (1.2–2.8)	1.9 (1.2–3.1)
Use of injectable contraceptives	*n* = 288		*n* = 141			
No	135	95.7	277	96.2	1	1
Yes	6	4.3	11	3.8	1.1 (0.4–2.9)	1.0 (0.3–3.0)
Condom use	*n* = 139		*n* = 284			
Yes	12	8.6	69	24.3	1	1
No	127	91.4	215	75.7	3.3 (1.8–6.4)	3.2 (1.5–6.8)

^a^OR: Non-adjusted Odd Ratio

^b^ORa: Adjusted Odd Ratio for residence area and HPV infection

### Sexual behavior

In the bivariate analysis (Table 5), the age at first sexual intercourse <18 years (OR vs. ≥18 years =2.4) and having had ≥2 lifetime sexual partners (OR vs. 1 sexual partner =2.1) were significant risk factors for cervical cancer. Women who had sexual intercourse during menstruation had an increased risk for cervical cancer (OR = 4.3). Compared to women whose husband had one sexual partner, women whose husbands had two sexual partners and more had an increased risk for cervical cancer (OR = 3.3). Compared to women who always washed the genital area after sexual intercourse, women who never or sometimes washed the genital area after intercourse had an increased risk for cervical cancer (OR = 17.1). History of sexually transmitted infections was significantly associated with an increase risk of cervical cancer (OR = 4.6). After adjustment for residence area and HPV infection, all associations observed in the bivariate analyses remained significant except the association between cervical cancer and the number of lifetime sexual partners for women.

**Table 5:** Women sexual characteristics, Sexually Transmitted Infections and hygienic practices among 144 cases of cervical cancer and 288 controls

	Cases		Controls		OR^a^ (95% CI)	ORa^b^ (95% CI)
	No.	(%)	No.	(%)		
Age at first intercourse (years)	*n* = 132		*n* = 272			
≥ 18	59	44.7	170	62.5	1	1
< 18	73	55.3	102	37.5	2.4 (1.5–3.9)	2.3 (1.3–3.9)
No. of lifetime sexual partners	*n* = 135		*n* = 272			
1	107	79.2	241	88.6	1	1
≥ 2	28	20.8	31	11.4	2.1 (1.2–3.8)	1.7 (0.8–3.4)
Sexual intercourse during menstruation	*n* = 138		*n* = 280			
No	99	71.7	256	91.4	1	1
Yes	39	28.3	24	8.6	4.3 (2.4–7.9)	4.7 (2.3–9.7)
Husband’s number of sexual partners	*n* = 129		*n* = 269			
One	67	51.9	210	78.1	1	1
Two and more	62	48.1	59	21.9	3.3 (2.1–5.2)	2.8 (1.3–6.3)
History of Sexually Transmitted Infections	*n* = 138		*n* = 270			
No	123	89.1	262	97.0	1	1
Yes	15	10.9	8	3.0	4.6 (1.6–12.9)	6.7 (1.9–23.6)
History of herpes infection	*n* = 139		*n* = 273			
No	133	95.7	257	94.1	1	1
Yes	6	4.3	16	5.9	0.7 (0.3–1.9)	0.6 (0.3–2.1)
History of condyloma infection	*n* = 139		*n* = 281			
No	133	95.7	274	97.5	1	1
Yes	6	4.3	7	2.5	1.6 (0.5–4.9)	1.4 (0.4–5.2)
Genital washing after intercourse	*n* = 138		*n* = 270			
Always	51	37.0	241	89.3	1	1
Sometimes or never	87	63.0	29	10.7	17.1 (8.2–35.4)	18.6 (7.9–43.7)

^a^OR: Non-adjusted Odd Ratio

^b^ORa: Adjusted Odd Ratio for residence area and HPV infection

### Other factors

Regarding smoking status, only 2.1% of cases and 1.8% of controls had a smoking history ≤1 packets/years (OR vs. never smoking = 1.4; 95%CI = 0.3–6.0) while 2.8% and 0.3% of controls had a smoking >1 packets/years (OR vs. never smoking = 8.3; 95%CI = 0.9–74.9). We found no statistically significant associations between cervical cancer and family history of cancer.

### Multivariate analysis

In the multivariate analysis (Table 6), an increased risk for cervical cancer was found among non-educated women (OR vs. educated =4.6), women with 4 and more pregnancies (OR vs. ≤3 pregnancies = 1.7), women who had sexual intercourse during menstruation (OR = 9.9), women whose husband had multiple sexual partners (OR vs. women whose husband had one sexual partner =2.9) and women who had a history of sexually transmitted infections (OR = 11.1).

**Table 6:** Determinants of invasive cervical cancer: multivariate analysis with conditional logistic regression among 144 cases of cervical cancer and 288 controls

	Cases		Cases Controls		OR (95% CI)
	No.	(%)	No.	(%)	
Education	*n* = 134		*n* = 273		
Educated	21	15.7	118	43.2	1
Non-educated	113	84.3	155	56.8	4.6 (1.8–11.6)
Husband’s number of sexual partners	*n* = 129		*n* = 269		
One	67	51.9	210	78.1	1
Two and more	62	48.1	59	21.9	2.9 (1.3–6.7)
No. of pregnancies	*n* = 141		*n* = 276		
≤3	40	28.4	119	43.1	1
>3	101	71.6	157	56.9	1.7 (1.1–4.3)
Sexual intercourse during menstruation					
No	99	71.7	256	91.4	1
Yes	39	28.3	24	8.6	9.9 (2.8–35.6)
History of Sexually Transmitted Infections	*n* = 138		*n* = 270		
No	123	89.1	262	97.0	1
Yes	15	10.9	8	3.0	11.1 (1.7–71.5)

Multicentre case-control study; Morocco; 2009–2012

## Discussion

This case-control study provides information on the risk factors for cervical cancer and the infection of HPV in Morocco. The results confirm the finding of the investigators indicating that HPV DNA is present in the vast majority of cervical cancers (92.5%) [12, 13]. Odd ratios linked to HPV sub-types demonstrated very strong associations for total HPV (OR = 39.3), HPV 16 (OR = 49.3), HPV 18 (OR = 31.7), and multiple infections (OR = 13.8). In this study, the other identified determinants of invasive cervical cancer were high parity, low educational level, husband’s multiple sexual partners, sexual intercourse during menstruation and history of sexually transmitted infections.

### HPV and cervical cancer

All HPV infections in cervical cancer cases were a high risk. This finding is similar to that found in other worldwide [3] or Moroccan studies [9]. HPV 16 in single or multiple infections was by far the most common type since it has been found in 85.0% of cervical cancer cases. Overall, these findings suggest that the distribution of HPV sub-types in cervical cancer in Morocco resembles more closely to the distribution found in similar studies in Europe and North America than in the rest of the African continent, where HPV 16 accounts for less than 50% of HPV positivity [13, 14]. This difference can be explained by the geographic location, socioeconomic and sociocultural characteristics of Morocco. Indeed, it is a country located in North Africa, it has maritime border with Europe. Its cultural transition is mainly marked by Westernization. There was a variation in HPV-specific prevalence between different histological cancer types, HPV 16 was found in all adenocarcinoma and in 79.2% of squamous cell carcinoma; this result is similar to that found in North Africa and South America where HPV 16 was found in 72% of the adenocarcinoma cases [15]. However, worldwide HPV 16 was identified in 55.2% of squamous cell carcinoma and in 31.3% of adenocarcinoma [13]. Only 6.0% of squamous cell carcinoma cases and no adenocarcinoma cases were infected with HPV 18, which disagrees with other studies in which it is reported that throughout the world, HPV 18 is present in 12.3% of squamous cell carcinoma [13] and 37.7–39.0% in adenocarcinoma [13, 15]. The fraction of squamous cell carcinoma attributable to HPV 16 and 18 was 74.8% while that for adenocarcinoma was 85.8%, almost the same as reported worldwide [16].

The frequency of HPV infection (13.9%) among control women in Morocco was intermediate, between the low prevalence found in Europe [17] and the high prevalence found in sub-Saharan Africa [18] and Latin America [19]. These differences in the prevalence of HPV in control women are consistent with the wide variations observed in cervical cancer incidence rates. HPV 16 was also by far the most common type among control women (51.3% of HPV-positive control women) which is in concordance with the worldwide distribution of HPV sub-types in cytologically normal women reported by the International Agency for Research on Cancer HPV in 2005 [20].

### Determinants of cervical cancer

Because of the infection by high risk HPV is a necessary, but not a sufficient, cause for cervical cancer [3], it has been assumed that other factors contribute to modulate the risk of transition from cervical HPV infection to cervical cancer.

Our results suggest also that high parity increases the risk of cervical cancer. The explanatory mechanism is not clear [21] but a known suggestion says that pregnancy could influence HPV effects on the cervical epithelium through immunologic or hormone-dependent mechanisms.

The number of sexual partners was not a determinant in the development of cervical cancer, as reported in other World populations. This could be explained by the fact that most of the women in our study reported a unique lifetime sexual partner. In Morocco, polygamy is not a frequent practice among women, in this study 79.2% of the cases and 88.6% of controls women said that they were monogamous. However, polygamy is a common practice among men in our study (48.1% of cases’ sexual partners and 21.9% of controls’ sexual partners). Geographic clusters of cervical and penile cancers [22], as well as elevated rates of cervical cancer among the wives of men with penile cancer [23, 24] raised the suspicion that a “male factor” might be important. This notion was supported by a follow-up study in which the wives of men previously married to cervical cancer patients were found to have elevated rates of cervical cancer compared to control wives [25].

Women who most likely belong to lower social classes, in which education and incomes are quiet low, are mainly subjects to cervical cancer [26, 27]. Also, cervical HPV infections appear to have historically been more prevalent in women of lower educational levels [28, 29].

In this study, sexual intercourse during menstruation was a determinant of cervical cancer. The explanatory mechanism is not clear, but a related finding was noted in Sichuan, China, where a strong protection was observed in women who abstained from sexual intercourse during menses [30].

In our study, history of sexually transmitted infections represents a risk factor for cervical cancer in Moroccan women. This result is consistent with other studies. Indeed, markers of exposure to other sexually transmitted infections have been found associated with cervical cancer repeatedly [31, 32].

Our study has many strong methodological points. We conducted a multicenter case-control study which improves the representativeness of cases of cervical cancer of the study compared to the cases of cervical cancer in Morocco. The two cancer centers of Rabat and Casablanca represent the first and largest oncology centers in Morocco. Before 2007, they were the only public centers for oncology in Morocco [33]. In our study we included only incident cases of cervical cancer which allowed us to avoid a possible selective survival bias. Also, controls were individually matched on age with cases which gave rise to the advantage of making the cases and controls more comparable except for the disease. At the same time, this matching allowed us controlling the effects of age. In our analysis, the matching was taken into account by using conditional logistic regression [34].

There are some limitations in the design of our study. The protocol was intended to identify a group of controls individually matched by age groups to the age distribution of the cases. The selection of cases was made in cancer centers; which attracts patients from a wide, ill-defined reference population. This is particularly true for cases of cervical cancer, which are referred for radiotherapy from distant areas in Morocco. Controls were derived from public outpatient gynecological hospitals and communal health-care centers with gynecological unit resulting in a group of predominantly urban residency compared with the cervical cancer cases. In Morocco, the residence area is an important indicator of the socioeconomic and educational levels [35] Women living in rural areas are of lower socioeconomic and lower educational levels than those residents in urban areas [35]. So, the residence area was carefully selected from all available indicators of socio-economic status as the strongest discriminants between cases and controls and adjustments were made in the analyses by including systematically as confounder variable the residence area.

## Conclusions

In Morocco, HPV is the central cause of the cervical cancer cases. Currently, two HPV vaccines are available, a bivalent vaccine against HPV 16 and 18, and a tetravalent vaccine against HPV 6, 11, 16, and 18. In Morocco, the vaccine in theory could prevent 86.5% of cervical cancer cases. Despite the development of vaccination, screening programs remain a mainstay in the secondary prevention of cervical cancer. Identifying the factors, other than HPV, that contribute to the development of cervical cancer is essential.

The results of this study indicate that certain factors, such as low educational level, male sexual behavior, multiparity, sexual intercourse during menstruation, and history of sexually transmitted infections are the main lifestyle risk factors for cervical cancer in Morocco. This information could be used to install a program based on health education and to prioritize limited screening and treatment services to the benefit of women who have these specific characteristics putting them at an increased risk of cervical cancer.

## Abbreviations

HPV: Human Papillomavirus
HPV-DNA: Human Papilomavirus - DNA
OR: Odds Ratio
PCR: Polymerase Chain Reaction
